# Plasma metabonomics of classical swine fever virus-infected pigs

**DOI:** 10.3389/fvets.2023.1171750

**Published:** 2023-12-06

**Authors:** Jiedan Liao, Wenshuo Hu, Weijun Wang, Xinyan Wang, Shu Yu, Xinni Niu, Wenhui Zhu, Bolun Zhou, Yiwan Song, Weijun Zeng, Zhimin Lu, Jinding Chen

**Affiliations:** ^1^School of Life Science and Engineering, Foshan University, Foshan, China; ^2^College of Veterinary Medicine, South China Agricultural University, Guangzhou, China

**Keywords:** classical swine fever virus, metabonomics, metabolic pathway, tricarboxylic acid cycle, heat map

## Abstract

Classical swine fever (CSF) is an infectious disease caused by Classical swine fever virus (CSFV), which is characterized by depression, high fever, extensive skin bleeding, leukopenia, anorexia, alternating constipation, and diarrhea. Hemorrhagic infarction of the spleen is the main characteristic pathological change following CSFV infection. Large-scale outbreaks of CSF are rare in China and are mainly distributed regionally. The clinical symptoms of CSF are not obvious, and show variation from typical to atypical symptoms, which makes diagnosis based on clinical symptoms and pathology challenging. In recent years, the incidence of CSF-immunized pig farms in China has increased and new CSFV gene subtypes have appeared, posing new challenges to the prevention and control of CSF in China. Changes in metabolites caused by viral infection reflect the pathogenic process. Metabonomics can reveal the trace metabolites of organisms; however, plasma metabonomics of CSFV-infected pigs have rarely been investigated. Therefore, we used an established pig CSFV infection model to study changes in plasma metabolites. The results showed significant differences in forty-five plasma metabolites at different time periods after CSFV infection in pigs, with an increase in twenty-five metabolites and a decrease in twenty metabolites. These changed metabolites were mainly attributed to the tricarboxylic acid cycle, amino acid cycle, sugar metabolism, and fat metabolism. Thirteen metabolic pathways changed significantly in CSFV-infected pigs, including tricarboxylic acid cycle, inositol phosphate metabolism, glycine, serine and threonine metabolism,lysine degradation, alanine, aspartate and glutamic acid metabolism, pantothenate and CoA biosynthesis, β-alanine metabolism, lysine degradation, arginine and proline metabolism, glycerolipid metabolism, phenylalanine metabolism, arachidonic acid metabolism, linoleic acid metabolism. Among these, changes in fatty acid biosynthesis and metabolism occurred at all time periods post-infection. These results indicate that CSFV infection in pigs could seriously alter metabolic pathways.

## Introduction

Viruses can use host cell glycolysis, the tricarboxylic acid cycle, lipid metabolism, amino acid metabolism, and nucleotide metabolism to promote their own replication in the host (1). Recent studies have shown that human cytomegalovirus (HCMV) can alter many aspects of host cell metabolism, including metabolic pathways that are crucial for viral replication (2). Hepatitis C virus can utilize host glucose and induce the dysregulation of lipid biosynthesis after establishing infection (3). When the body is infected with a virus, the cellular metabolic pathway changes to facilitate viral replication (4). In a study on hepatitis B virus (HBV) transgenic mice, the HBV antigen was found to interfere with lipid metabolism in the liver (5). The HBV viral envelope was found to absorb external lipids from host cells to meet the needs of the viral lipid membranes (6).

Metabonomics is an approach that relies on trace metabolites for comprehensive, systematic, quantitative, and high-throughput analysis and evaluation of living organisms in response to a stimulus (such as pathogen infection), which shows great potential in the discovery of disease biomarkers (7, 8). The complex relationship between pathogens and hosts can be better explained using genomics, proteomics, and metabonomics. Metabolic analysis techniques such as gas chromatography–mass spectrometry (GC–MS), liquid chromatography-mass spectrometry, and nuclear magnetic resonance combined with multivariate statistical analyses are widely used to provide valuable information such as the metabolic disorders induced by stimulation after virus infection (9). The analysis of key metabolites in body fluids has become an important part of improving the diagnosis, treatment, and prognosis of various diseases (10).

Classical swine fever (CSF) is a highly contagious infectious disease in pigs and wild boars caused by Classical swine fever virus (CSFV). CSF is characterized by immunosuppression such as systemic hemorrhage, septicemia, and leukopenia (10). Our previous study showed that CSFV infection can cause a large reduction in leukocyte subsets, accompanied by changes in liver and kidney metabolic indices, suggesting liver and kidney damage. Pathological and histopathological studies have confirmed that some tissues and organs of CSFV-infected pigs exhibit inflammation and lesions. Based on these findings, we speculated that CSFV infection can cause changes in plasma metabolites. To more deeply understand the pathogenesis of CSF and explore whether plasma metabolites after CSFV infection can serve as potential biomarkers with diagnostic value, in the present study, we performed plasma metabonomics to quantitatively compare the metabolites of uninfected and CSFV-infected pigs at different time points post-infection. This study could help to identify new CSFV biomarkers, and provides a reference for the pathogenesis of CSF and the establishment of new clinical disease diagnosis methods.

## Materials and methods

### Virus

Blood poison of the CSFV Shimen strain was used as the infection source, which is preserved at the Department of Microbiology and Immunology, College of Veterinary Medicine, South China Agricultural University.

### Antibody

The anti-CSFV E2 monoclonal antibody (9011) was purchased from JBT Company of Korea. Alexa 488-labeled goat anti-mouse IgG (A0428) secondary antibody was obtained from Beyotime.

### Virus proliferation and titer determination

When the PK-15 cell monolayer grew to 80–90% fusion, the cells were washed twice with Dulbecco’s modified Eagle medium (DMEM) without serum and antibiotics, inoculated with CSFV-infected blood, and adsorbed at 37°C for 2 h. The culture bottle was gently shaken every 20 min to ensure that the CSFV solution would be evenly spread across the surface of the cell monolayer. After adsorption, the cells were washed twice with DMEM cell culture solution without serum and antibiotics, incubated with the cell maintenance culture solution, and cultured at 37°C in a 5% CO_2_ incubator. After 72 h, the virus solution was harvested and stored at −80°C for later use.

The cells were passaged in a 96-well cell culture plate and inoculated with CSFV when the cell monolayer grew to 80–90% confluence. The virus solution obtained was diluted to 10^−10^ with DMEM cell culture solution without serum and antibiotics, and then the CSFV solution of each gradient was inoculated into four wells of the cell culture plate at 0.1 mL/well; blank wells were used as a control. After inoculation, the cells were cultured continuously for 2 days, and then the cell culture plate was removed for indirect immunofluorescence detection to calculate the CSFV infection titer. In detail, the culture medium was carefully removed, washed with phosphate-buffered saline (PBS) three times, −20°C pre-cooled absolute ethanol was added, and the cells were fixed at −20°C for 30 min. The absolute ethanol was discarded and the cells were washed with PBS three times. The cells were incubated with CSFV E2 protein antibody (diluted with 0.01 mol/L PBS; 1:100 dilution) at 37°C for 1 h. After washing with PBS three times, Alexa 488-labeled goat anti-mouse IgG (diluted with 0.01 mol/L PBS; 1:400 dilution) was added in the dark and incubated at 37°C for 1 h. The cells were washed again with PBS three times, and the cell culture plate was observed under a fluorescence microscope. The blank control was set to establish the presence of fluorescent foci as CSFV-positive and the absence of fluorescent foci as CSFV-negative. The number of wells with fluorescent foci was recorded and the CSFV infection titer was calculated as the median tissue culture infectious dose (TCID_50_)/mL.

### Animal grouping and infection

Ten Tibetan miniature pigs aged 4 months and weighing about 20–25 kg were purchased from the Experimental Animal Center of Southern Medical University. Reverse transcription-polymerase chain reaction and enzyme-linked immunoassay were used to detect nucleic acids and antibodies against common infectious diseases in pigs, including CSFV, pseudorabies virus, porcine respiratory and reproductive syndrome virus, porcine circovirus type II, porcine parvovirus, and foot-and-mouth disease virus type O. Pigs with negative indices for all pathogens were selected as experimental pigs.

Ten Tibetan miniature pigs that were confirmed to be completely negative for the above pathogens were randomly divided into two groups and fed separately, with five pigs in the CSFV-infected group (three females, two males) and five pigs in the mock-infected group (three females, two males). The pigs were observed and treated with parasite drugs for 1 week. Before the experiment, body temperature was measured at a fixed time for several consecutive days, and the average value was taken as the basal body temperature. Pigs in good health were selected for formal experiments.

Before blood collection, the pigs were fasted for 12 h and allowed to drink freely. On the day of CSFV infection, blood samples were collected, and five pigs were injected with CSFV Shimen strain-infected blood at 1 × 10^5^ TCID_50_/head into the neck muscles, and the other five mock-infected were injected with an equal volume of normal saline in the neck muscles. The infection day was set as 0 days post-inoculation (DPI), and the first day after infection was 1 DPI. Blood samples were collected from mock- and CSFV-infected pigs at a fixed time every alternate day.

This experiment was approved by the Committee of Experimental Animal Ethics of South China Agricultural University, and all procedures were strictly in accordance with the requirements of animal experiments.

### Plasma sample collection

The results of the preliminary experiment showed that there was no significant difference in plasma metabolites between the mock-infected groups. In order to get closer to the blood collection points of the CSFV-infected group, two time points mg1 (1DPI)and mg2 (3DPI)were selected for the mock-infected groups. The blood sample (4 mL) was centrifuged at 4000 rpm for 10 min, and the supernatant was collected and stored at −80°C for testing. Blood samples were taken at two points for the mock-infected group mg1 and mg2, whereas blood samples were taken at four points in the CSFV-infected group: cs-1DPI, cs-3DPI, cs-5DPI, and cs-7DPI for the detection and comparison of metabolites.

### Metabolite extraction and sample derivatization

Twenty microliters of thawed plasma samples and 80 μL of ice-cold methanol were sequentially added to an EP tube. The mixture was vortexed for 30 s, stored at −20°C for 1 h, and then centrifuged at 4°C for 15 min at 16,000 *g*. The supernatant (80 μL) was transferred to 10 μL of internal standard (0.02 mg/mL of L-phenylalanine-^13^C_9_-^15^N, 0.05 mg/mL of galactitol, L-leucine-^13^C_6_ and L-isoleucine-^13^C_6_-^15^N, 0.1 mg/mL of L-valine-^13^C_5_-^15^N and L-alanine-^13^C_3_-^15^N). The mixture was dried under mild nitrogen flow and 30 μL of 20 mg/mL methoxyamine hydrochloride (anhydrous pyridine) was added to the dried residue. The mixture was vortexed for 30 s and then incubated at 37°C for 90 min. Thirty microliters of N,O-bis (trimethylsilyl)trifluoroacetamide (containing 1% trimethylchlorosilane) was added to the mixture and derivatized at 70°C for 60 min.

### GC–MS detection

Samples were analyzed by an Agilent 7890A gas chromatograph and Agilent 5975C mass spectrometer. The chromatographic conditions were as follows: HP-5MS (30 m × 0.25 mm × 0.25 μm) chromatographic column, helium (>99.999%) as the carrier gas, and the column flow rate was 1 mL/min. The loading volume was 1 μL and the solvent delay time was 6 min. The temperature was maintained at 70°C for 2 min, increased to 160°C at a rate of 6°C/min, increased to 240°C at a rate of 10°C/min, increased to 300°C at a rate of 20°C/min, and finally maintained at 300°C for 6 min. The MS conditions were as follows: interface temperature, 250°C; fourth-stage rod temperature, 290°C; and ion source temperature, 230°C. The ionization voltage was 70 eV and full-scan mode (m/z: 50–600) was used to collect data.

### Data preprocessing and statistical analysis

To correct the mass spectrum response, the concentration and total peak area of each sample were normalized. The peak table (matrix X) file was imported into Simca-P V11.0 (Umetrics AB, Sweden) for centralization, and the data were averaged to the unit variance. After standardization, multivariate statistical analysis was performed, including principal component analysis (PCA) and partial least-squares discriminant analysis (PLS-DA). The quality of the model is described by R^2^X (the explanatory rate of the X model) or R^2^Y (the observed quantity of Y is the explanatory rate of differences between groups), and Q^2^ (the predictive rate). R^2^X (PCA) or R^2^Y (PLS-DA) is defined as the proportion of variance in the data explained by the model and indicates the goodness of fit. Q^2^ is defined as the proportion of variance in the data that the model can predict, and indicates the predictability of the current model, which is calculated through the cross-validation process. To avoid model overfitting, the default 7-round cross-validation was performed, which provided the best number of principal components as determined in Simca-P. The R^2^X, R^2^Y, and Q^2^ values were used as indicators to evaluate the main symbolic parameters of the pattern recognition model. The statistical significance of differential metabolites between the infected and uninfected groups was evaluated by Student’s *t*-test (*p* < 0.05).

## Results

### Data inspection

**Figure 1:**
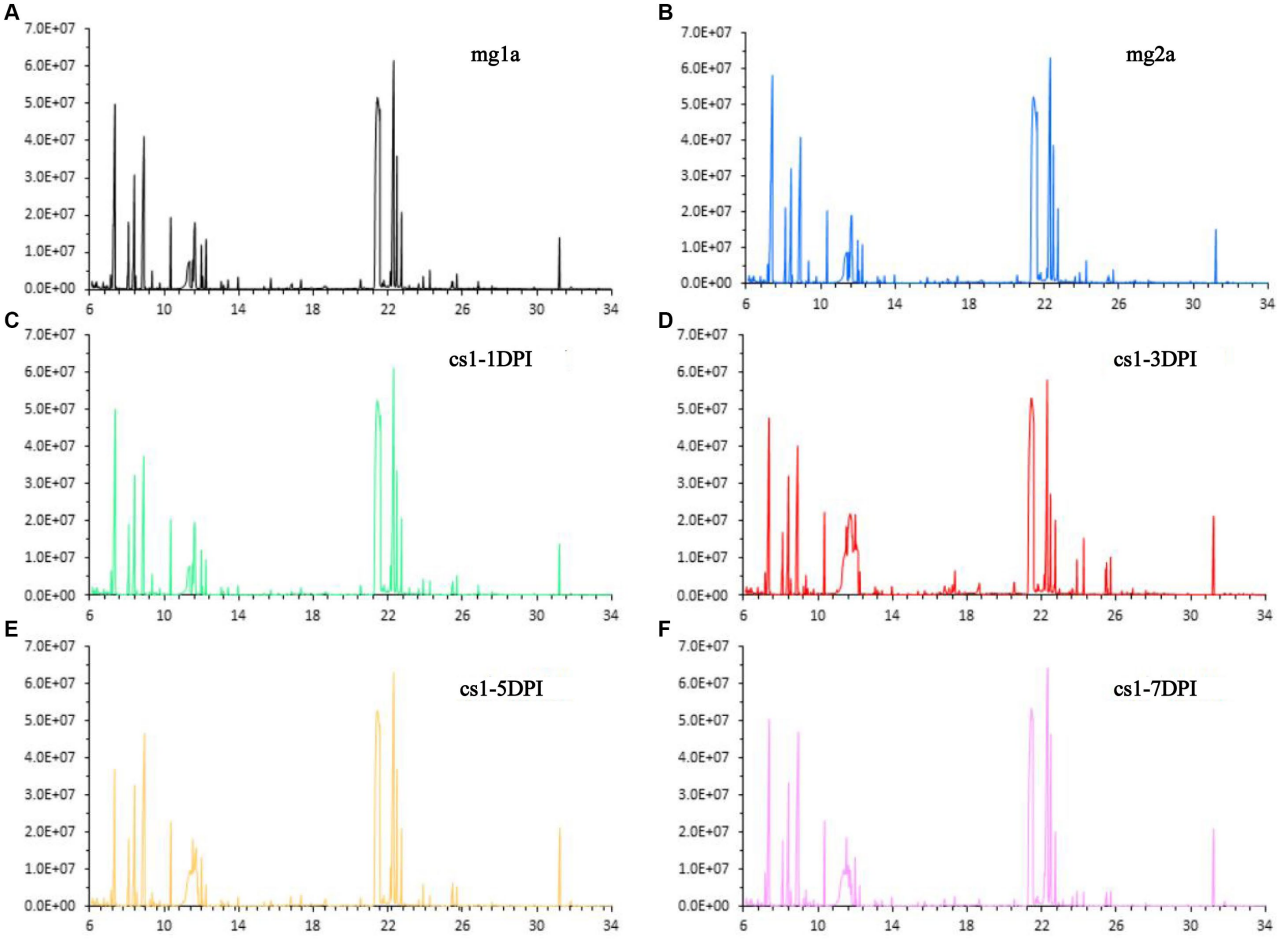
Representative total ion chromatograms. **(A)** Mock-infected a-1DPI. **(B)** Mock-infected a-3DPI. **(C)** CSFV-infected 1-1DPI. **(D)** CSFV-infected 1-3DPI. **(E)** CSFV-infected 1-5DPI. **(F)** CSFV-infected 1-7DPI.

The data of CSFV titration for the same strain have been published previously (11). Stability of the detection method is an important prerequisite for obtaining reliable metabolomic data. First, a group of plasma samples infected with CSFV-infected and two groups of plasma samples from the mock-infected were subjected to total ion chromatography (TIC). The TIC of all samples showed characteristics of a strong signal, large peak capacity, and good retention time reproducibility, which indicated that the GC–MS method adopted in this study has good reproducibility and stability, and could be used for the high-throughput analysis of subsequent samples. Representative TIC results for each group are shown in Figure 1.

### Multivariate data analysis

#### PCA

**Figure 2:**
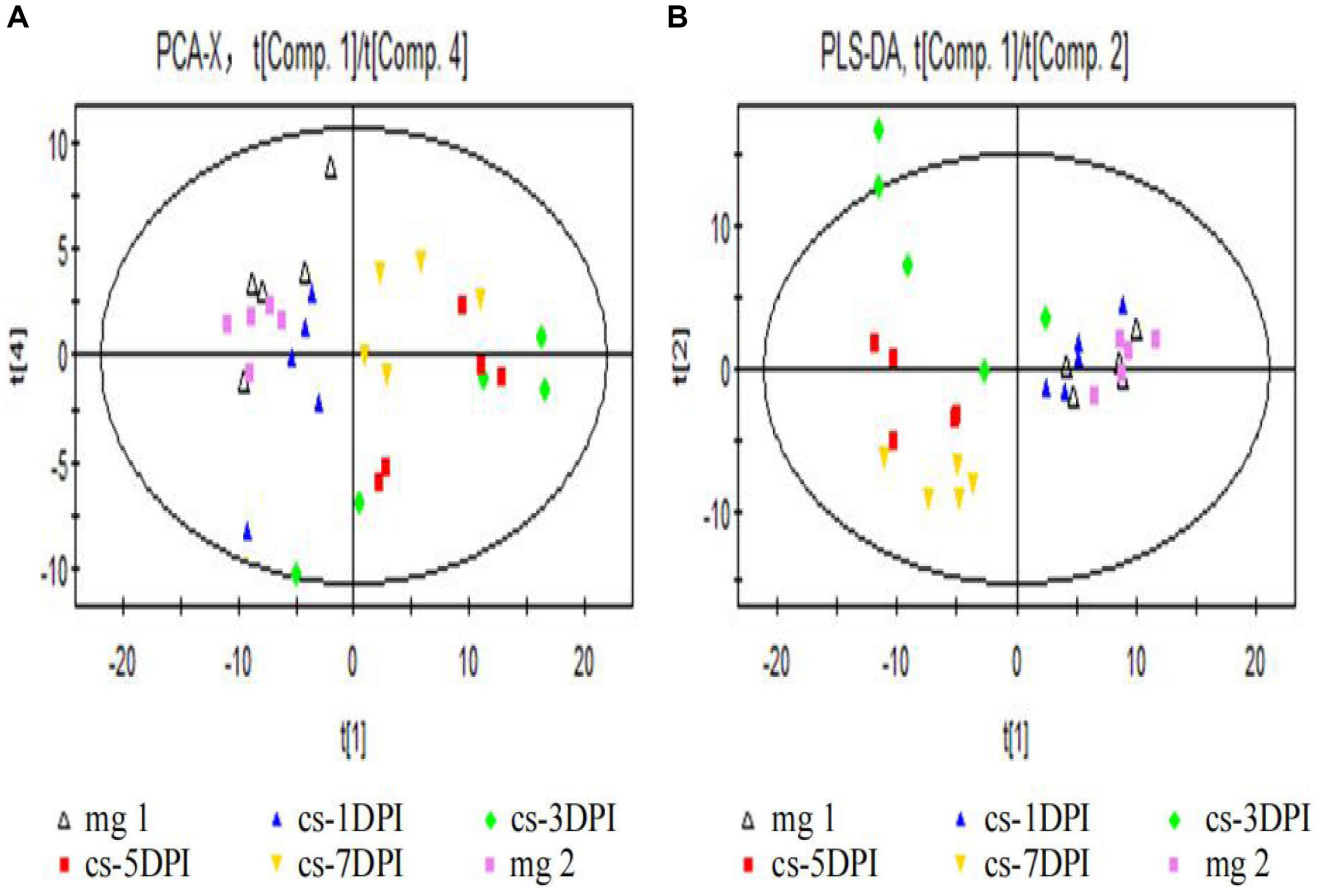
Overall PCA **(A)** and PLS-DA **(B)** scores between mock- and CSFV-infected groups.In all images, mg1 and mg2 represented Mock-infected groups (mg1-1DPI、mg2-3DPI), cs represented CSFV-infected groups.

First, we used PCA to reveal the clustering and metabolic pattern differences between CSFV-infected and mock-infected samples. The main parameter used to judge the quality of the PCA model was the cumulative explanatory rate R^2^X. If R^2^X is greater than 0.5, the model can be judged to be reliable. A PCA score chart is shown in Figure 2A, and four principal components were obtained. The cumulative explanatory rate of the model was R^2^X = 0.58, Q^2^ = 0.337. In general, an R^2^X value greater than 0.5 indicates that the model is reliable; thus, the obtained PCA model can be reliably used to reflect the metabolic differences between the two groups of samples. All samples were within the 95% confidence interval (Hotelling’s T2 ellipse), and there were no abnormal samples. From the PCA score plot (Figure 2A, the abscissa indicates the first principal component PC1, which is represented by t[1], and the ordinate indicates the 4th principal component PC4, which is represented by t[4]), there was no significant separation between the plasma samples of the mock-infected mg1 and mg2, whereas there was significant separation between the plasma samples of the CSFV-infected cs-1DPI, cs-3DPI, cs-5DPI and cs-7DPI on PC1 and PC4, which indicated that significant metabolic changes occurred over time after infection.

#### PLS-da

To eliminate the influence of background noise, PLS-DA-supervised multidimensional statistical analysis was used to analyze the models of the two groups of samples. This type of analysis is mainly used to eliminate intragroup variation, thus highlighting the differences between the groups. Seven principal components were obtained in this study: R^2^X = 0.687, R^2^Y = 0.781, and Q^2^ = 0.272. The score chart is shown in Figure 2B (the abscissa indicates the first principal component PC1, which is represented by t[1], and the ordinate represents the second principal component PC2, represented by t[2]). Among them, mock-infected mg1, mg2 and cs-1DPI samples are on the right side of PC1 (represented by t[1]), while cs-5DPI and cs-7DPI samples are on the left side of PC1, and five samples in the cs-3DPI are in transition. The intra-group variation of these five samples was very large, suggesting that the cs-3DPI group is in a drastic change stage. The model explanation rate R^2^Y showed that the PLS-DA model can explain the differences among the five groups of samples. There was no significant spectral separation on PCA and PLS-DA between the mock-infected groups mg1 and mg2, indicating that there were no significant metabolic differences between the groups. In order to get close to the stress response of the animals sampled in CSFV-infected group, metabolite differences in the mock-infected mg2 were compared with other time points in the CSFV-infected group.

### PCA and PLS-DA scores of CSFV- and mock-infected groups at different times

#### Day one

**Figure 3:**
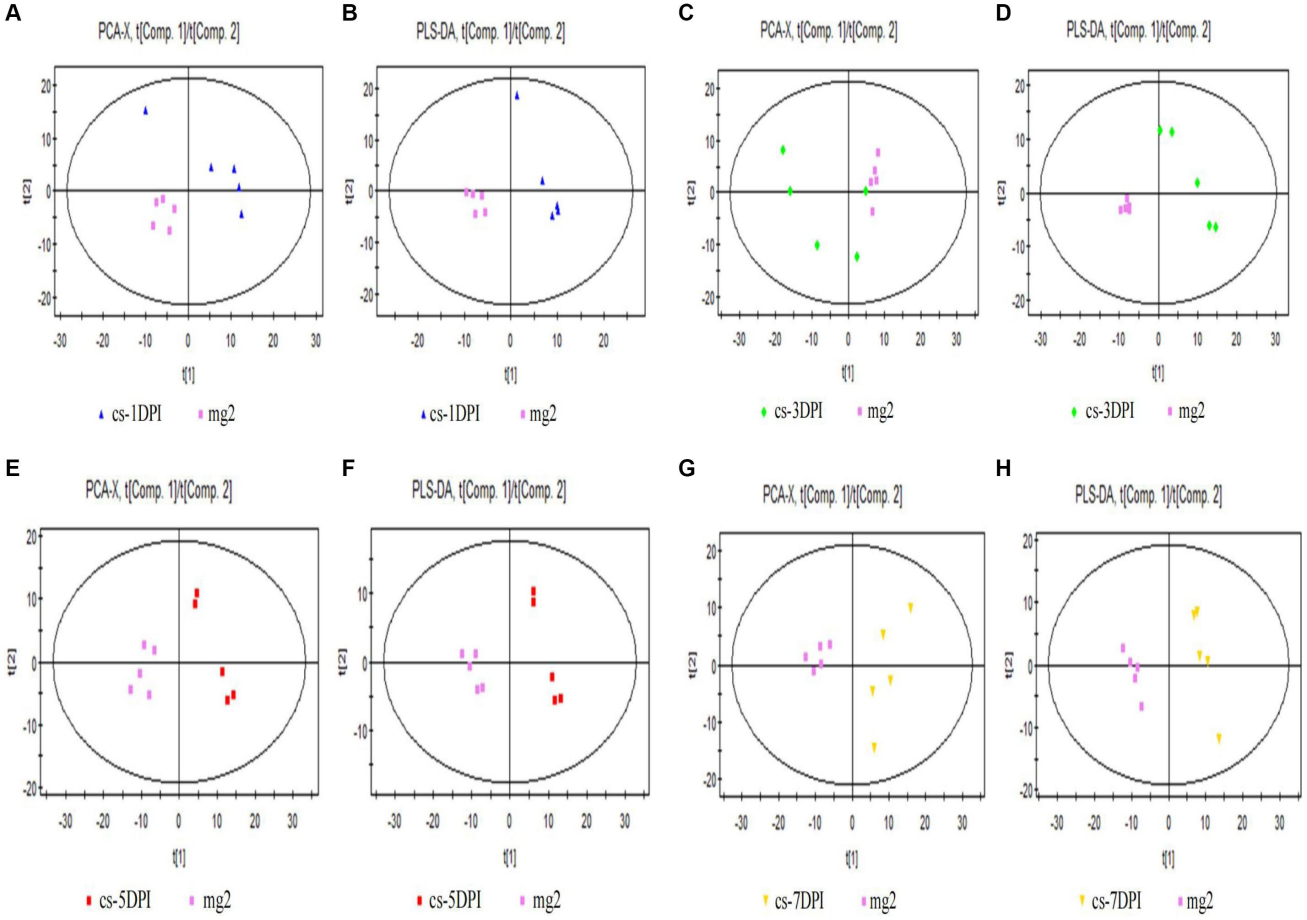
Overall PCA and PLS-DA score plots of CSFV-infected and mg2 on 1DPI **(A,B)**, 3DPI **(C,D)**, 5DPI **(E,F)**, and 7DPI **(G,H)**, respectively.

The PCA score plot for mock- and CSFV-infected samples at 1 DPI is shown in Figure 3A, with two principal components obtained. The cumulative explanatory rates of the model were R^2^X = 0.486 and Q^2^ = 0.0127. Therefore, this PCA model can reliably explain the metabolic differences between the two groups of samples. The cs-1DPI and mg2 showed obvious separation, which indicated clear metabolic differences between the two groups.

Significant differences were also observed between these two groups in the PLS-DA score chart. Three principal components were obtained: R^2^X = 0.562, R^2^Y = 0.998, and Q^2^ = 0.809. The score chart is shown in Figure 3B. Both the model explanation rate R^2^Y and the prediction rate Q^2^ were very high, indicating that the PLS-DA model can explain and predict the differences between the two groups of samples.

#### Day three

The PCA score plot for mock- and CSFV-infected samples at 3 DPI is shown in Figure 3C, with two principal components obtained. The cumulative explanatory rates of the model were R^2^X = 0.578 and Q^2^ = 0.197. Therefore, this PCA model can reliably explain the metabolic differences between the two groups of samples. The cs-3DPI and mg2 showed significant separation, which indicated clear differences between the two groups of samples.

There were also significant differences (spectral separation) in PLS-DA scores between these two groups. Two principal components were obtained: R^2^X = 0.548, R^2^Y = 0.987, and Q^2^ = 0.873. The score chart is shown in Figure 3D. Both the model explanation rate R^2^Y and the prediction rate Q^2^ were very high, indicating that the PLS-DA model can explain and predict the differences between the two groups of samples.

#### Day five

The PCA score plot for mock- and CSFV-infected samples at 5 DPI is shown in the left panel of Figure 3E, with two principal components obtained. The cumulative explanatory rates of the model were R^2^X = 0.565 and Q^2^ = 0.239. Therefore, this PCA model can reliably explain the metabolic differences between the two groups of samples. Groups cs-5DPI and mg2 are in two distinct positions, indicating a significant difference between the two groups of samples.

There were also significant differences (spectral separation) in PLS-DA scores between these two groups. Two principal components were obtained: R^2^X = 0.552, R^2^Y = 0.99, and Q^2^ = 0.924. The score chart is shown in Figure 3F. Both the model explanation rate R^2^Y and the prediction rate Q^2^ were very high (close to 1), which indicated that the PLS-DA model can be used to explain and predict the differences between the two groups of samples very reliably.

#### Day seven

The PCA score plot for mock- and CSFV-infected samples at 7 DPI is shown in Figure 3G, with two principal components obtained. The cumulative explanatory rate of the model was R^2^X = 0.58, Q^2^ = 0.204. Therefore, this PCA model can reliably explain the metabolic differences between the two groups of samples. Groups cs-7DPI and mg2 were in two different positions, indicating a significant difference between the two groups.

There were also significant differences (spectral separation) in PLS-DA scores between these two groups. Two principal components were obtained: R^2^X = 0.558, R^2^Y = 0.993, and Q^2^ = 0.957. The score chart is shown in Figure 3H. Both the model explanation rate R^2^Y and the prediction rate Q^2^ were very high (close to 1), which indicated that the PLS-DA model can explain and predict the differences between the two groups of samples well.

### Pathway analysis of differential metabolites between CSFV- and mock-infected groups at different times

#### Day one

##### Differential metabolites

When searching for differential metabolites, the significance between mock- and CSFV-infected groups was examined using Student’s *t*-test (judged at *p* < 0.05). The first principal component variable importance in projection (VIP) value (threshold >1) of the PLS-DA model was used to identify differentially expressed metabolites. For qualitative assessment of metabolic changes, we first searched for a self-built reference material database, including Fiehn GC/MS Metabolomics RTL Library, Golm Metabolome Database, and NIST commercial database. The data of metabolites are presented in Table 1. A total of thirty-nine different substances were screened and identified in this study, including twenty metabolites with decreased abundance and nineteen metabolites with increased abundance after infection.

**Table 1:** The differences of metabolites between CSFV- and mock-infected groups at 1 day post-infection.

No.	Metabolites	VIP	*p* value	FC(cs-1DPI/mg2)	HMDB	KEGG	Pathway (KEGG)
1	2-ketoglutaric acid	1.72	9.16E-04	−0.36	HMDB00208	C00026	Citrate cycle (TCA cycle)
2	fumaric acid	1.54	1.37E-02	−0.26	HMDB00134	C00122	Citrate cycle(TCA cycle);
							Alanine, aspartate and glutamate metabolism
3	glutamic acid	1.44	1.70E-02	−0.71	HMDB00148	C00025	Alanine, aspartate and glutamate metabolism; Arginine and proline metabolism;
							Taurine and hypotaurine metabolism
4	4-hydroxyproline	1.68	1.55E-03	−0.62	HMDB00725	C01157	Arginine and proline metabolism
5	threonic acid	1.29	4.73E-02	0.85	HMDB00943	C01620	Ascorbate and aldarate metabolism
6	mannose	1.31	3.48E-02	1.15	HMDB00169	C00159	Fructose and mannose metabolism;
							Amino sugar and nucleotide sugar metabolism
7	pyroglutamic acid	1.28	4.06E-02	−0.5	HMDB00267	C01879	Glutathione metabolism
8	glycerol-3-phosphate	1.48	1.81E-02	−0.94	HMDB00126	C00093	Glycerolipid metabolism;
							Glycerophospholipid metabolism
9	glycine	1.46	1.86E-02	−0.32	HMDB00123	C00037	Glycine,serine and threonine metabolism;
							Lysine degradation
10	myo-inositol	1.31	3.55E-02	−2.11	HMDB00211	C00137	Inositol phosphate metabolism
11	myo-inositol-1-phosphate	1.36	3.60E-02	−0.98	HMDB00213	C04006	Inositol phosphate metabolism
12	5-aminovaleric acid	1.69	2.13E-03	−0.68	HMDB03355	C00431	Lysine degradation
13	lysine	1.36	3.06E-02	1.08	HMDB00182	C00047	Lysine degradation;
							Biotin metabolism
14	ribitol	1.31	4.12E-02	−0.49	HMDB00508	C00474	Pentose and glucuronate interconversions
15	lanosterol	1.4	2.23E-02	−0.48	HMDB01251	C01724	Steroid biosynthesis
16	hypotaurine	1.42	2.01E-02	−0.39	HMDB00965	C00519	Taurine and hypotaurine metabolism
17	2-aminobutyric acid	1.74	6.72E-04	1.94	HMDB00452	C02356	fatty acid, monoamino
18	2-hydroxybutyric acid	1.74	6.97E-04	1.82	HMDB00008	C05984	fatty acid, monohydroxy
19	11-eicosenoic acid	1.78	3.05E-04	1.29	HMDB34296		Long chain fatty acid
20	cis-10-heptadecenoic acid	1.56	5.90E-03	0.91	HMDB33188	C14416	Long chain fatty acid
21	cis-11,14-eicosadienoic acid	1.51	1.05E-02	0.86			Long chain fatty acid
22	lauric acid	1.37	2.57E-02	0.94	HMDB00638	C02679	Long chain fatty acid
23	linoleic acid	1.4	2.60E-02	0.88	HMDB00673	C01595	Long chain fatty acid
24	myristic acid	1.32	3.63E-02	0.99	HMDB00806	C06424	Long chain fatty acid
25	oleic acid	1.33	3.66E-02	1.36	HMDB00207	C00712	Long chain fatty acid
26	palmitic acid	1.33	3.82E-02	0.66	HMDB00220	C00249	Long chain fatty acid
27	palmitoleic acid	1.29	4.05E-02	1.85	HMDB03229	C08362	Long chain fatty acid
28	stearic acid	1.3	4.35E-02	0.46	HMDB00827	C01530	Long chain fatty acid
29	trans-oleic acid	1.27	4.99E-02	0.81	HMDB00573	C00712	Long chain fatty acid
30	2,4-dihydroxybutyric acid	1.75	5.15E-04	0.2	HMDB00360		
31	4-deoxyerythronic acid	1.71	1.06E-03	0.53	HMDB00498		
32	4-deoxythreonic acid	1.7	1.55E-03	0.67	HMDB02453		
33	aminomalonic acid	1.59	5.45E-03	−0.82	HMDB01147	C00872	
34	erythritol	1.5	1.09E-02	−1.18	HMDB02994	C00503	
35	erythronic acid	1.47	1.36E-02	−0.23	HMDB00613		
36	galacturonic acid	1.45	1.52E-02	−0.32	HMDB02545	C08348	
37	glucuronic acid	1.45	1.69E-02	−0.61	HMDB00127	C00191	
38	indole-3-propionic acid	1.46	2.14E-02	−2.21	HMDB02302		
39	lyxose	1.34	3.29E-02	−1.04	HMDB03402	C08348	

As shown in Table 1, for the cs-1 DPI, the concentrations of fatty acids significantly increased, whereas the concentrations of intermediates of lipid metabolism, including glycerol-3-phosphate (phosphoglycerol dehydrogenase) and myo-inositol-1-phosphate (inositol −1- phosphoglycerol), significantly decreased. These findings indicated that the anabolism of fatty acids at 1 DPI significantly enhanced, resulting in a large consumption of two precursor materials used for lipid synthesis.

##### Pathway analysis

**Figure 4:**
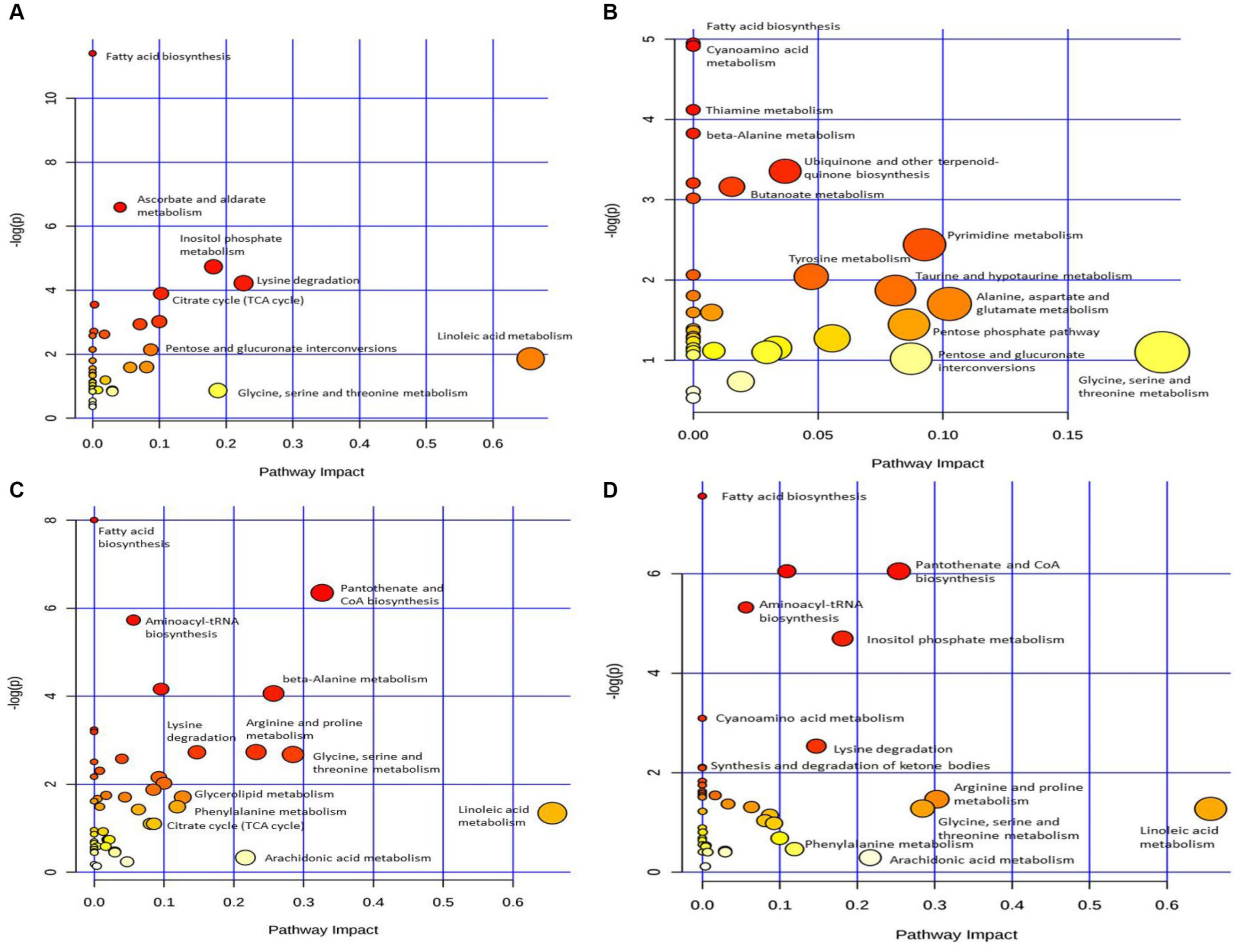
Pathway analysis of differential metabolism between CSFV-infected and mg2 on 1DPI **(A)**, 3DPI **(B)**, 5DPI **(C)**, and 7DPI **(D)**, respectively.

To explore the metabolic pathways that underwent significant changes in the plasma after CSFV infection, we used the software MetaboAnalyst to perform a preliminary pathway analysis of the two groups of different metabolites. As shown in Figure 4A, CSFV infection at 1 DPI caused changes in eight metabolic pathways: fatty acid biosynthesis, Ascorbate and aldarate metabolism, inositol phosphate metabolism, lysine degradation, tricarboxylic acid cycle, pentose and glucuronate interconversion, linoleic acid metabolism, and glycine, serine and threonine metabolism. There were significant changes in tricarboxylic acid cycle, inositol phosphate metabolism, glycine, serine and threonine metabolism, lysine degradation (impact >0.1).The detailed pathway data are shown in Table 2, where “total” indicates the total number of metabolites in the pathway, “hits” indicates the number of differential metabolites in this pathway, “–log(p)” indicates the ordinate value of Figure 4A, and “impact” indicates the abscissa value of Figure 4A.

**Table 2:** Pathway analysis between CSFV- and mock-infected groups at 1 day post-infection.

No.	Pathway	Total	Expected	Hits	Raw p	#NAME?	Holm adjust	FDR	Impact
1	Phenylalanine metabolism	45	0.31782	3	0.003458	5.667	0.27666	0.27666	0.06073
2	Vitamin B6 metabolism	32	0.22601	2	0.020567	3.8841	1	0.82267	0.1642
3	Fatty acid metabolism	50	0.35314	2	0.047156	3.0543	1	1	0.02959
4	Tyrosine metabolism	76	0.53677	2	0.098602	2.3167	1	1	0.00458
5	Citrate cycle (TCA cycle)	20	0.14125	1	0.13266	2.02	1	1	0.01446
6	Alanine, aspartate and glutamate metabolism	24	0.16951	1	0.15712	1.8508	1	1	0
7	Fatty acid elongation in mitochondria	27	0.19069	1	0.17504	1.7428	1	1	0
8	Propanoate metabolism	35	0.2472	1	0.22108	1.5092	1	1	0.00134
9	Inositol phosphate metabolism	39	0.27545	1	0.24319	1.4139	1	1	0
10	Butanoate metabolism	40	0.28251	1	0.24862	1.3918	1	1	0.01774
11	Valine, leucine and isoleucine degradation	40	0.28251	1	0.24862	1.3918	1	1	0.02232
12	Ascorbate and aldarate metabolism	45	0.31782	1	0.27524	1.2901	1	1	0.03322
13	Lysine degradation	47	0.33195	1	0.28564	1.253	1	1	0.06505
14	Fatty acid biosynthesis	49	0.34607	1	0.29589	1.2178	1	1	0
15	Starch and sucrose metabolism	50	0.35314	1	0.30097	1.2007	1	1	0
16	Glyoxylate and dicarboxylate metabolism	50	0.35314	1	0.30097	1.2007	1	1	0
17	Pentose and glucuronate interconversions	53	0.37432	1	0.31599	1.152	1	1	0.08723
18	Aminoacyl-tRNA biosynthesis	75	0.52971	1	0.41723	0.87412	1	1	0
19	Amino sugar and nucleotide sugar metabolism	88	0.62152	1	0.47023	0.75452	1	1	0
20	Purine metabolism	92	0.64977	1	0.48561	0.72235	1	1	0.00577

#### Day three

##### Differential metabolites

The data of metabolites that changed at 3 DPI are presented in Table 3. A total of twenty-six different substances were screened and identified, including eight metabolites with decreased abundance and eighteen metabolites with increased abundance at 3 DPI.

**Table 3:** The differences of metabolites between CSFV- and mock-infected groups at 3 days post-infection.

No.	Metabolites	VIP	*p* value	FC (cs-3DPI/mg2)	HMDB	KEGG	Pathway (KEGG)
1	3-hydroxybutyric acid	1.56	2.78E-04	1.18	HMDB00357	C01089	ketone bodies Alanine, aspartate and glutamate metabolism;
2	gama-aminobutyric acid	1.44	3.33E-02	0.95	HMDB00112	C00334	Arginine and proline metabolism;
							beta-Alanine metabolism
3	alanine	1.37	5.46E-03	−0.59	METPA0179	C01401	Alanine, aspartate and glutamate metabolism;
							Cysteine and methionine metabolism;
							Taurine and hypotaurine metabolism
4	2-aminobutyric acid	1.2	2.33E-02	2.87	HMDB00452	C02356	fatty acid, monoamino
5	2-hydroxybutyric acid	1.33	8.50E-03	3.27	HMDB00008	C05984	fatty acid, monohydroxy
6	3-hydroxyadipic acid	1.1	3.97E-02	1.5	HMDB00345	C00156	fatty acid, monohydroxy
7	arachidic acid	1.14	4.47E-02	0.94	HMDB02212	C06425	Long chain fatty acid
8	cis-11,14-eicosadienoic acid	1.35	1.35E-02	1.59			Long chain fatty acid
9	myristic acid	1.38	2.83E-03	1.37	HMDB00806	C06424	Long chain fatty acid
10	oleic acid	1.28	7.11E-04	1.89	HMDB00207	C00712	Long chain fatty acid
11	palmitoleic acid	1.61	6.27E-03	2.94	HMDB03229	C08362	Long chain fatty acid
12	mannose	1.37	5.17E-03	0.97	HMDB00169	C00159	Fructose and mannose metabolism;
							Amino sugar and nucleotide sugar metabolism
13	glycine	1.24	1.15E-03	−0.74	HMDB00123	C00037	Glycine, serine and threonine metabolism;
							Lysine degradation
14	ethanolamine	1.2	9.59E-03	0.74	HMDB00149	C00189	Phosphonate and phosphinate metabolism;
							Glycerophospholipid metabolism
15	cytosine	1.16	2.38E-03	1.97	HMDB00630	C00380	Pyrimidine metabolism
16	uracil	1.12	4.07E-02	1.8	HMDB00300	C00106	Pyrimidine metabolism;
							beta-Alanine metabolism
17	beta-sitosterol	1.23	0.0136	0.82	HMDB00852	C01753	Steroid biosynthesis
18	2-hydroxyisovaleric acid	1.22	0.0304	0.84	HMDB00407	C00072	Synthesis and degradation of ketone bodies
19	hypotaurine	1.33	0.00251	−0.82	HMDB00965	C00519	Taurine and hypotaurine metabolism
20	4-hydroxyphenyllactic acid	1.36	0.0471	−0.46	HMDB00755	C03672	Tyrosine metabolism
21	tyrosine	1.11	6.24E-03	−1.68	HMDB00158	C00082	Tyrosine metabolism;
							Phenylalanine metabolism
22	dehydroascorbic acid	1.46	3.14E-05	−0.92	HMDB01264	C00425	Cyanoamino acid metabolism
23	gluconic acid	1.18	3.00E-02	2.06	HMDB00625	C00257	Degradation of aromatic compounds
24	glucuronic acid	1.17	4.59E-02	2.14	HMDB00127	C00191	Fatty acid
25	gycerol-2-phosphate	1.6	9.38E-03	−0.37			Lipid metabolism

##### Pathway analysis

Figure 4B shows that CSFV infection at 3 DPI caused changes in fatty acid biosynthesis, cyanoamino acid metabolism, thiamine metabolism, β-alanine metabolism, ubiquinone and other terpenoid quinone biosynthesis, butanoate metabolism, tyrosine metabolism, pyrimidine metabolism, taurne and hypotaurine metabolism, pentose phosphate pathway, pentose and glucuronate interconversions, alanine, aspartate and glutamic acid metabolism, glycine, serine and threonine metabolism. Among these pathways, there are significant changes in alanine, aspartate and glutamic acid metabolism, glycine, serine and threonine metabolism (impact >0.1).The metabolic pathway diagram analysis is presented in Table 4.

**Table 4:** Pathway analysis between CSFV- and mock-infected groups at 3 days post-infection.

No.	Pathway	Total	Expected	Hits	Raw p	#NAME?	Holm adjust	FDR	Impact
1	Fatty acid biosynthesis	49	0.40715	3	0.007086	4.9496	0.56688	0.2937	0
2	Cyanoamino acid metabolism	16	0.13295	2	0.007343	4.9141	0.58006	0.2937	0
3	Thiamine metabolism	24	0.19942	2	0.016229	4.121	1	0.43277	0
4	beta-Alanine metabolism	28	0.23265	2	0.02179	3.8263	1	0.4358	0
5	Ubiquinone and other terpenoid-quinone biosynthesis	36	0.29913	2	0.034906	3.3551	1	0.48424	0.0368
6	Nitrogen metabolism	39	0.32405	2	0.040451	3.2077	1	0.48424	0
7	Butanoate metabolism	40	0.33236	2	0.042371	3.1613	1	0.48424	0.01547
8	Synthesis and degradation of ketone bodies	6	0.049855	1	0.04888	3.0184	1	0.4888	0
9	Pyrimidine metabolism	60	0.49855	2	0.08714	2.4402	1	0.77457	0.09259
10	Aminoacyl-tRNA biosynthesis	75	0.62318	2	0.12696	2.0638	1	0.91719	0
11	Tyrosine metabolism	76	0.63149	2	0.12976	2.042	1	0.91719	0.04724
12	Taurine and hypotaurine metabolism	20	0.16618	1	0.15426	1.8691	1	0.91719	0.08094
13	Amino sugar and nucleotide sugar metabolism	88	0.7312	2	0.16443	1.8053	1	0.91719	0
14	Alanine, aspartate and glutamate metabolism	24	0.19942	1	0.18227	1.7023	1	0.91719	0.10256
15	Pantothenate and CoA biosynthesis	27	0.22435	1	0.20269	1.5961	1	0.91719	0
16	Pentose phosphate pathway	32	0.26589	1	0.23566	1.4454	1	0.91719	0.08639
17	Methane metabolism	34	0.28251	1	0.24848	1.3924	1	0.91719	0
18	Propanoate metabolism	35	0.29082	1	0.25482	1.3672	1	0.91719	0
19	Glutathione metabolism	38	0.31575	1	0.27352	1.2964	1	0.91719	0
20	Inositol phosphate metabolism	39	0.32405	1	0.27965	1.2742	1	0.91719	0
21	Glycerophospholipid metabolism	39	0.32405	1	0.27965	1.2742	1	0.91719	0.05562
22	Galactose metabolism	41	0.34067	1	0.29177	1.2318	1	0.91719	0
23	Phenylalanine metabolism	45	0.37391	1	0.31543	1.1538	1	0.91719	0
24	Ascorbate and aldarate metabolism	45	0.37391	1	0.31543	1.1538	1	0.91719	0.03322
25	Lysine degradation	47	0.39053	1	0.32697	1.1179	1	0.91719	0
26	Primary bile acid biosynthesis	47	0.39053	1	0.32697	1.1179	1	0.91719	0.00822
27	Fructose and mannose metabolism	48	0.39884	1	0.33268	1.1006	1	0.91719	0.02948
28	Glycine, serine and threonine metabolism	48	0.39884	1	0.33268	1.1006	1	0.91719	0.18774
29	Starch and sucrose metabolism	50	0.41545	1	0.34395	1.0673	1	0.91719	0
30	Pentose and glucuronate interconversions	53	0.44038	1	0.36051	1.0202	1	0.93036	0.08723
31	Arginine and proline metabolism	77	0.6398	1	0.47946	0.7351	1	1	0.01905
32	Purine metabolism	92	0.76444	1	0.54278	0.61106	1	1	0
33	Porphyrin and chlorophyll metabolism	104	0.86415	1	0.58809	0.53087	1	1	0

#### Day five

##### Differential metabolites

The data of metabolites that changed at 5 DPI are presented in Table 5. A total of sixty-five different substances were screened and identified, including twenty metabolites with decreased abundance and forty-five metabolites with increased abundance at 5 DPI.

**Table 5:** The differences of metabolites between CSFV- and mock-infected groups at 5 days post-infection.

No.	Metabolites	VIP	value of p	FC(cs-5DPI/mg2)	HMDB	KEGG	Pathway (KEGG)
1	2-ketoglutaric acid	1.02	4.18E-02	0.34	HMDB00208	C00026	Citrate cycle (TCA cycle)
2	malic acid	1.24	1.00E-02	−0.44	HMDB00744	C00711	Citrate cycle (TCA cycle)
3	glucose-6-phosphate	1.01	2.80E-02	−2	HMDB01401	C00092	Glycolysis / Gluconeogenesis;
4	glyceric acid	1.15	1.99E-02	0.51	HMDB00139	C00258	Pentose phosphate pathway;
							Glycine, serine and threonine metabolism;
							Glycerolipid metabolism
5	3-hydroxybutyric acid	1.36	1.21E-03	1.06	HMDB00357	C01089	ketone bodies
6	methylmaleic acid	1.04	4.02E-02	0.39	HMDB00634	C02226	2-oxocarboxylic acid metabolism
7	beta-alanine	1.32	2.70E-03	0.96	HMDB00056	C00099	Alanine and aspartate metabolism
8	alanine	1.43	3.69E-04	−0.64	METPA0179	C01401	Alanine, aspartate and glutamate metabolism;
							Cysteine and methionine metabolism;
							Taurine and hypotaurine metabolism
9	4-hydroxyproline	1.46	1.48E-04	−1.33	HMDB00725	C01157	Arginine and proline metabolism
10	creatinine	1.25	5.59E-03	0.59	HMDB00562	C00791	Arginine and proline metabolism
11	proline	1.52	1.33E-05	−1.38	HMDB00162	C00148	Arginine and proline metabolism
12	putrescine	1.17	1.47E-02	0.8	HMDB01414	C00134	Arginine and proline metabolism
13	threonic acid	1.13	2.15E-02	1.01	HMDB00943	C01620	Ascorbate and aldarate metabolism
14	pantothenic acid	1.29	3.62E-03	−0.53	HMDB00210	C00864	beta-Alanine metabolism;
							Pantothenate and CoA biosynthesis
15	hexanoic acid	1.14	1.95E-02	0.49	HMDB00535	C01585	fatty acid
16	2-aminobutyric acid	1.41	3.93E-04	2.96	HMDB00452	C02356	fatty acid, monoamino
17	2-hydroxybutyric acid	1.38	7.02E-04	3.66	HMDB00008	C05984	fatty acid, monohydroxy
18	3-hydroxyadipic acid	1.09	2.73E-02	1.63	HMDB00345	C11118	fatty acid, monohydroxy
19	3-hydroxyisovaleric acid	1.24	7.81E-03	1.52	HMDB00754	C16884	fatty acid, monohydroxy
20	glycolic acid	1.44	1.59E-04	0.36	HMDB00115	C00160	fatty acid, monohydroxy
21	1-oleoylglycerol	1.13	2.34E-02	2.04			lipid
22	2-stearoylglycerol	1.4	1.04E-03	−0.48			lipid
23	11-eicosenoic acid	1.41	3.13E-04	2.21	HMDB34296		Long chain fatty acid
24	arachidic acid	1.19	1.12E-02	0.95	HMDB02212	C06425	Long chain fatty acid
25	arachidonic acid	1.18	2.07E-02	−0.53	HMDB01043	C00219	Long chain fatty acid
26	cis-10-heptadecenoic acid	1.36	1.19E-03	1.52	HMDB33188	C14416	Long chain fatty acid
27	cis-11,14-eicosadienoic acid	1.15	1.74E-02	1.31			Long chain fatty acid
28	lauric acid	1.2	1.09E-02	1.31	HMDB00638	C02679	Long chain fatty acid
29	linoleic acid	1.19	1.15E-02	1.41	HMDB00673	C01595	Long chain fatty acid
30	myristic acid	1.4	4.41E-04	1.34	HMDB00806	C06424	Long chain fatty acid
31	oleic acid	1.43	2.38E-04	2.11	HMDB00207	C00712	Long chain fatty acid
32	palmitelaidic acid	1.2	1.06E-02	1.24	HMDB12328		Long chain fatty acid
33	palmitic acid	1.38	6.48E-04	0.95	HMDB00220	C00249	Long chain fatty acid
34	palmitoleic acid	1.42	2.75E-04	2.73	HMDB03229	C08362	Long chain fatty acid
35	stearic acid	1.31	2.50E-03	0.53	HMDB00827	C01530	Long chain fatty acid
36	trans-oleic acid	1.41	2.74E-02	1.29	HMDB00573	C00712	Long chain fatty acid
37	mannose	1.43	2.83E-04	1.86	HMDB00169	C00159	Fructose and mannose metabolism;
							Amino sugar and nucleotide sugar metabolism
38	glycerol-3-phosphate	1.37	8.88E-04	−1.04	HMDB00126	C00093	Glycerolipid metabolism; Glycerophospholipid metabolism
39	phosphoethanolamine	1.18	1.18E-02	−0.6	HMDB00224	C00346	Glycerophospholipid metabolism
40	threonine	1.12	3.46E-04	−0.5	HMDB00167	C00188	Glycine, serine and threonine metabolism
41	glycine	1.47	9.63E-05	−0.99	HMDB00123	C00037	Glycine, serine and threonine metabolism;
							Lysine degradation
42	myo-inositol-1-phosphate	1.56	3.46E-07	−1.77	HMDB00213	C04006	Inositol phosphate metabolism
43	pipecolinic acid	1.17	1.32E-02	−2.49	HMDB00070	C00408	Lysine degradation
44	lysine	1.11	2.34E-02	0.8	HMDB00182	C00047	Lysine degradation;
							Biotin metabolism
45	phenylalanine	1.38	2.07E-03	1.77	HMDB00159	C00079	Phenylalanine metabolism
46	ethanolamine	1.1	2.51E-02	0.46	HMDB00149	C00189	Phosphonate and phosphinate metabolism;
							Glycerophospholipid metabolism
47	inosine	1.52	8.10E-06	−2.16	HMDB00195	C00294	Purine metabolism
48	cytosine	1.32	2.28E-03	0.68	HMDB00630	C00380	Pyrimidine metabolism
49	uracil	1.25	8.45E-03	0.62	HMDB00300	C00106	Pyrimidine metabolism;
							beta-Alanine metabolism
50	beta-sitosterol	1.42	4.55E-04	1.4	HMDB00852	C01753	Steroid biosynthesis
51	cholesterol	1.45	1.11E-04	1.07	HMDB00067	C00187	Steroid biosynthesis;
							Steroid degradation
52	2-hydroxyisovaleric acid	1.13	1.96E-02	1.19	HMDB00407		Synthesis and degradation of ketone bodies
53	hypotaurine	1.5	2.40E-05	−1.64	HMDB00965	C00519	Taurine and hypotaurine metabolism
54	tyrosine	1.09	6.06E-03	−0.96	HMDB00158	C00082	Tyrosine metabolism;
							Phenylalanine metabolism
55	2-ketoisovaleric acid	1.17	1.25E-02	0.55	HMDB00019	C00141	Valine, leucine and isoleucine degradation
56	1-octadecanol	1.2	1.18E-02	0.53	HMDB02350	D01924	
57	2,4-dihydroxybutyric acid	1.16	1.82E-02	0.46	HMDB00360		
58	4-deoxyerythronic acid	1.17	1.75E-02	1.55	HMDB00498		
59	4-deoxythreonic acid	1.3	2.82E-03	2.85	HMDB02453		
60	campesterol	1.35	1.42E-03	1.21	HMDB02869	C01789	
61	dehydroascorbic acid	1.31	2.55E-03	−0.49	HMDB01264	C00425	
62	erythronic acid	1.16	1.91E-02	1.19	HMDB00613		
63	gycerol-2-phosphate	1.49	1.86E-05	−0.55			
64	indole-3-propionic acid	1.18	1.63E-02	−2.29	HMDB02302		
65	N-acetylgalactosamine	1.19	1.09E-02	0.81	HMDB00212	C01074	
66	N-acetylgalactosamine	1.43	3.66E-04	0.99	HMDB00212	C01074	

##### Pathway analysis

Figure 4C shows that infection with CSFV at 5 DPI caused changes in twelve metabolic pathways, including fatty acid biosynthesis, pantothenate and CoA biosynthesis, aminoacyl-tRNA biosynthesis,β-alanine metabolism, lysine degradation, arginine and proline metabolism, glycine, serine and threonine metabolism, glycerolipid metabolism, phenylalanine metabolism, citrate cycle,arachidonic acid metabolism, linoleic acid metabolism. In the above metabolic pathways, except Fatty acid biosynthesis, aminoacyl-tRNA biosynthesis and citrate cycle, other nine metabolic pathways are significant differences (impact >0.1). Pathway analysis data are shown in Table 6.

**Table 6:** Pathway analysis between CSFV- and mock-infected groups at 5 days post-infection.

No.	Pathway	Total	Expected	Hits	Raw p	#NAME?	Holm adjust	FDR	Impact
1	Fatty acid biosynthesis	49	0.97715	6	0.000334	8.0051	0.0267	0.0267	0
2	Pantothenate and CoA biosynthesis	27	0.53843	4	0.001745	6.3509	0.13787	0.069808	0.32666
3	Aminoacyl-tRNA biosynthesis	75	1.4956	6	0.003244	5.7309	0.25304	0.086509	0.05634
4	beta-Alanine metabolism	28	0.55837	3	0.017189	4.0635	1	0.27503	0.25694
5	Cyanoamino acid metabolism	16	0.31907	2	0.03912	3.2411	1	0.4706	0
6	Nitrogen metabolism	39	0.77773	3	0.041178	3.1899	1	0.4706	0
7	Arginine and proline metabolism	77	1.5355	4	0.065147	2.7311	1	0.54264	0.23195
8	Lysine degradation	47	0.93727	3	0.065453	2.7264	1	0.54264	0.14725
9	Glycine, serine and threonine metabolism	48	0.95721	3	0.068868	2.6756	1	0.54264	0.28482
10	Glyoxylate and dicarboxylate metabolism	50	0.99709	3	0.075936	2.5779	1	0.54264	0.03977
11	Thiamine metabolism	24	0.4786	2	0.081397	2.5084	1	0.54264	0
12	Synthesis and degradation of ketone bodies	6	0.11965	1	0.11395	2.172	1	0.61716	0
13	Pyrimidine metabolism	60	1.1965	3	0.11572	2.1566	1	0.61716	0.09259
14	Propanoate metabolism	35	0.69796	2	0.15318	1.8761	1	0.69259	0.085
15	Glutathione metabolism	38	0.75779	2	0.17448	1.746	1	0.69259	0.01714
16	Inositol phosphate metabolism	39	0.77773	2	0.18168	1.7055	1	0.69259	0.0441
17	Glycerophospholipid metabolism	39	0.77773	2	0.18168	1.7055	1	0.69259	0.12691
18	Butanoate metabolism	40	0.79767	2	0.18893	1.6664	1	0.69259	0.0048
19	D-Glutamine and D-glutamate metabolism	11	0.21936	1	0.19912	1.6139	1	0.69259	0
20	Biotin metabolism	11	0.21936	1	0.19912	1.6139	1	0.69259	0
21	Ascorbate and aldarate metabolism	45	0.89738	2	0.22573	1.4884	1	0.72232	0.00802
22	Phenylalanine metabolism	45	0.89738	2	0.22573	1.4884	1	0.72232	0.11906
23	Primary bile acid biosynthesis	47	0.93727	2	0.24062	1.4246	1	0.74036	0.06346
24	Linoleic acid metabolism	15	0.29913	1	0.26143	1.3416	1	0.77461	0.65625
25	Taurine and hypotaurine metabolism	20	0.39884	1	0.33268	1.1006	1	0.91773	0.08094
26	Citrate cycle (TCA cycle)	20	0.39884	1	0.33268	1.1006	1	0.91773	0.08577
27	Alanine, aspartate and glutamate metabolism	24	0.4786	1	0.38479	0.95506	1	1	0
28	Sphingolipid metabolism	25	0.49855	1	0.39718	0.92336	1	1	0.01288
29	Fatty acid elongation in mitochondria	27	0.53843	1	0.42124	0.86456	1	1	0
30	Vitamin B6 metabolism	32	0.63814	1	0.47734	0.73953	1	1	0.01914
31	Glycerolipid metabolism	32	0.63814	1	0.47734	0.73953	1	1	0.0206
32	Pentose phosphate pathway	32	0.63814	1	0.47734	0.73953	1	1	0.02181
33	Methane metabolism	34	0.67802	1	0.49825	0.69665	1	1	0
34	Ubiquinone and other terpenoid-quinone biosynthesis	36	0.71791	1	0.51835	0.6571	1	1	0
35	Purine metabolism	92	1.8346	2	0.55456	0.58958	1	1	0.00425
36	Valine, leucine and isoleucine degradation	40	0.79767	1	0.55621	0.58661	1	1	0.01657
37	Galactose metabolism	41	0.81762	1	0.56521	0.57056	1	1	0
38	Histidine metabolism	44	0.87744	1	0.59115	0.52569	1	1	0
39	Porphyrin and chlorophyll metabolism	104	2.074	2	0.62258	0.47388	1	1	0
40	Fructose and mannose metabolism	48	0.95721	1	0.62339	0.47258	1	1	0.02948
41	Starch and sucrose metabolism	50	0.99709	1	0.63856	0.44853	1	1	0.00031
42	Fatty acid metabolism	50	0.99709	1	0.63856	0.44853	1	1	0.02959
43	Arachidonic acid metabolism	62	1.2364	1	0.7178	0.33156	1	1	0.21669
44	Tyrosine metabolism	76	1.5156	1	0.78892	0.23709	1	1	0.04724
45	Amino sugar and nucleotide sugar metabolism	88	1.7549	1	0.83566	0.17954	1	1	0

#### Day seven

##### Differential metabolites

The data of metabolites that changed at 7 DPI are presented in Table 7. A total of sixty-nine different substances were screened and identified, including twenty-six metabolites with decreased abundance and forty-three metabolites with increased abundance at 7 DPI.

**Table 7:** The differences of metabolites between CSFV- and mock-infected groups at 7 days post-infection.

No.	Metabolites	VIP	value of p	FC(cs-7DPI/mg2)	HMDB	KEGG	Pathway (KEGG)
1	glucose-6-phosphate	1.09	2.76E-02	−2.13	HMDB01401	C00092	Glycolysis / Gluconeogenesis;
2	ribose	1.07	3.68E-02	−0.4	HMDB00283	C00121	Pentose phosphate pathway
3	3-hydroxybutyric acid	1.25	6.72E-03	1.08	HMDB00357	C01089	ketone bodies
4	methylmaleic acid	1.07	3.61E-02	0.46	HMDB00634	C02226	2-oxocarboxylic acid metabolism
5	alanine	1.37	1.35E-03	−0.47	METPA0179	C01401	Alanine, aspartate and glutamate metabolism;
							Cysteine and methionine metabolism;
							Taurine and hypotaurine metabolism
6	4-hydroxyproline	1.5	4.94E-05	−1.51	HMDB00725	C01157	Arginine and proline metabolism
7	ornithine	1.33	2.57E-03	−1.18	HMDB00214	C00077	Arginine and proline metabolism
8	proline	1.53	1.81E-05	−1.16	HMDB00162	C00148	Arginine and proline metabolism
9	pantothenic acid	1.36	1.96E-03	−0.63	HMDB00210	C00864	beta-Alanine metabolism;
							Pantothenate and CoA biosynthesis
10	adipic acid	1.15	1.87E-02	0.66	HMDB00448	C06104	Caprolactam degradation
11	hexanoic acid	1.01	4.80E-02	0.58	HMDB00535	C01585	fatty acid
12	2-aminobutyric acid	1.23	9.21E-03	2.08	HMDB00452	C02356	fatty acid, monoamino
13	norleucine	1.17	1.83E-02	0.91	HMDB01645	C01933	fatty acid, monoamino
14	2-hydroxybutyric acid	1.33	2.81E-03	3.39	HMDB00008	C05984	fatty acid, monohydroxy
15	3-hydroxyisovaleric acid	1.3	3.77E-03	1.45	HMDB00754	C00159	fatty acid, monohydroxy
16	glycolic acid	1.41	6.25E-04	0.79	HMDB00115	C00160	fatty acid, monohydroxy
17	1-oleoylglycerol	1.53	1.32E-05	1.02			lipid
18	2-stearoylglycerol	1.32	4.13E-03	−0.28			lipid
19	11-eicosenoic acid	1.39	9.88E-04	1.9	HMDB34296	C00037	Long chain fatty acid
20	arachidic acid	1.35	1.94E-03	0.83	HMDB02212	C06425	Long chain fatty acid
21	arachidonic acid	1.37	1.26E-03	−0.69	HMDB01043	C00219	Long chain fatty acid
22	cis-10-heptadecenoic acid	1.4	7.18E-04	1.08	HMDB33188	C14416	Long chain fatty acid
23	cis-11,14-eicosadienoic acid	1.16	1.82E-02	0.88			Long chain fatty acid
24	lauric acid	1.1	2.61E-02	0.84	HMDB00638	C02679	Long chain fatty acid
25	linoleic acid	1.21	1.17E-02	1.14	HMDB00673	C01595	Long chain fatty acid
26	myristic acid	1.31	3.40E-03	0.96	HMDB00806	C06424	Long chain fatty acid
27	oleic acid	1.44	2.85E-04	1.94	HMDB00207	C00712	Long chain fatty acid
28	palmitelaidic acid	1.08	3.54E-02	0.59	HMDB12328	C14416	Long chain fatty acid
29	palmitic acid	1.38	1.08E-03	0.81	HMDB00220	C00249	Long chain fatty acid
30	palmitoleic acid	1.5	4.88E-05	2.39	HMDB03229	C08362	Long chain fatty acid
31	stearic acid	1.31	3.34E-03	0.5	HMDB00827	C01530	Long chain fatty acid
32	trans-oleic acid	1.48	7.54E-05	1.09	HMDB00573	C00712	Long chain fatty acid
33	mannose	1.55	4.56E-06	1.96	HMDB00169	C00159	Fructose and mannose metabolism;
							Amino sugar and nucleotide sugar metabolism
34	glycerol-3-phosphate	1.33	2.54E-03	−0.76	HMDB00126	C00093	Glycerolipid metabolism; Glycerophospholipid metabolism
35	threonine	1.16	2.60E-02	−0.55	HMDB00167	C00188	Glycine,serine and threonine metabolism
36	serine	1.37	1.46E-03	−0.64	HMDB00187	C00065	Glycine,serine and threonine metabolism;
							Cysteine and methionine metabolism
37	glycine	1.51	5.63E-05	−0.99	HMDB00123	C00037	Glycine,serine and threonine metabolism;
							Lysine degradation
38	myo-inositol	1.32	3.36E-03	−1.76	HMDB00211	C00137	Inositol phosphate metabolism
39	myo-inositol-1-phosphate	1.57	4.43E-07	−1.21	HMDB00213	C04006	Inositol phosphate metabolism
40	pipecolinic acid	1.26	6.89E-03	−2.92	HMDB00070	C00408	Lysine degradation
41	lysine	1.08	3.20E-02	0.81	HMDB00182	C00047	Lysine degradation;
							Biotin metabolism
42	arabitol	1.22	1.06E-02	−0.32	HMDB00568	C01904	Pentose and glucuronate interconversions
43	phenylalanine	1.33	2.85E-03	1.23	HMDB00159	C00079	Phenylalanine metabolism
44	inosine	1.54	9.37E-06	−2.07	HMDB00195	C00294	Purine metabolism
45	cytosine	1.02	4.86E-02	−0.93	HMDB00630	C00380	Pyrimidine metabolism
46	uracil	1.05	3.94E-02	0.5	HMDB00300	C00106	Pyrimidine metabolism;

							beta-Alanine metabolism
47	beta-sitosterol	1.18	1.44E-02	1.3	HMDB00852	C01753	Steroid biosynthesis
48	campesterol	1.28	5.32E-03	1.19	HMDB02869	C01789	Steroid biosynthesis
49	lanosterol	1.49	6.60E-05	1.7	HMDB01251	C01724	Steroid biosynthesis
50	cholesterol	1.53	1.58E-05	1.23	HMDB00067	C00187	Steroid biosynthesis;
							Steroid degradation
51	2-hydroxyisovaleric acid	1.16	1.93E-02	1.25	HMDB00407		Synthesis and degradation of ketone bodies
52	hypotaurine	1.53	1.64E-05	−1.91	HMDB00965	C00519	Taurine and hypotaurine metabolism
53	2-ethyl-3-hydroxypropionic acid	1.24	7.69E-03	0.63	HMDB00396		Valine, leucine and isoleucine degradation
54	valine	1.24	8.68E-03	0.54	HMDB00883	C00183	Valine, leucine and isoleucine degradation
55	3-methyl-2-ketovaleric acid	1.01	4.76E-02	0.73	HMDB00491	C03465	Valine, leucine and isoleucine degradation
56	2-ketoisovaleric acid	1.14	2.06E-02	0.8	HMDB00019	C00141	Valine, leucine and isoleucine degradation
57	isoleucine	1.03	4.50E-02	0.52	HMDB00172	C00407	Valine, leucine and isoleucine degradation
58	1-octadecanol	1.21	1.31E-02	0.58	HMDB02350		
59	1-propanamine	1.26	6.65E-03	0.25	HMDB34006		
60	2,4-dihydroxybutyric acid	1.32	3.30E-03	0.51	HMDB00360		
61	2-hydroxypyridine	1.23	1.05E-02	0.25	HMDB13751	C02502	
62	4-deoxythreonic acid	1.47	1.58E-04	1.92	HMDB02453		
63	aminomalonic acid	1.14	2.67E-02	−0.89	HMDB01147	C00872	
64	erythritol	1.34	2.32E-03	−1.08	HMDB02994	C00503	
65	galacturonic acid	1.19	1.59E-02	−0.27	HMDB02545	C08348	
66	glucuronic acid	1.2	1.50E-02	−0.47	HMDB00127	C00191	
67	gycerol-2-phosphate	1.33	2.73E-03	−0.3			
68	indole-3-propionic acid	1.24	9.31E-03	−3.11	HMDB02302		
69	N-acetylgalactosamine	1.43	3.66E-04	0.99	HMDB00212	C01074	

##### Pathway analysis

Figure 4D shows that 7 DPI of CSFV caused changes in twelve metabolic pathways, including fatty acid biosynthesis, pantothenate and CoA biosynthesis, aminoacyl-tRNA biosynthesis, inositol phosphate metabolism, cyanoamino acid metabolism, lysine degradation, synthesis and degradation of ketone bodies, arginine and proline metabolism, glycine, serine and threonine metabolism, linoleic acid metabolism, phenylalanine metabolism, and arachidonic acid metabolism. In addition to fatty acid biosynthesis, aminoacyl-tRNA biosynthesis and cyanoamino acid metabolism, the other nine metabolic pathways showed significant differences (impact >0.1).Pathway analysis data are shown in Table 8.

**Table 8:** Pathway analysis between CSFV- and mock-infected groups at 7 days post-infection.

No.	Pathway	Total	Expected	Hits	Raw p	#NAME?	Holm adjust	FDR	Impact
1	Fatty acid biosynthesis	49	1.0586	6	0.00052	7.5608	0.041636	0.041636	0
2	Pantothenate and CoA biosynthesis	27	0.5833	4	0.002355	6.0514	0.18601	0.06279	0.2538
3	Aminoacyl-tRNA biosynthesis	75	1.6203	6	0.004876	5.3234	0.37546	0.097521	0.05634
4	Inositol phosphate metabolism	39	0.84254	4	0.009115	4.6979	0.69272	0.14584	0.18113
5	Cyanoamino acid metabolism	16	0.34566	2	0.045284	3.0948	1	0.60378	0
6	Lysine degradation	47	1.0154	3	0.079277	2.5348	1	0.90602	0.14725
7	beta-Alanine metabolism	28	0.6049	2	0.12125	2.1099	1	1	0
8	Synthesis and degradation of ketone bodies	6	0.12962	1	0.12294	2.0961	1	1	0
9	D-Arginine and D-ornithine metabolism	8	0.17283	1	0.16053	1.8293	1	1	0
10	Propanoate metabolism	35	0.75613	2	0.17386	1.7495	1	1	0
11	Glutathione metabolism	38	0.82094	2	0.19743	1.6224	1	1	0
12	Nitrogen metabolism	39	0.84254	2	0.20538	1.5829	1	1	0
13	Valine, leucine and isoleucine degradation	40	0.86415	2	0.21337	1.5447	1	1	0.01657
14	Biotin metabolism	11	0.23764	1	0.21396	1.542	1	1	0
15	Galactose metabolism	41	0.88575	2	0.22139	1.5078	1	1	0
16	Arginine and proline metabolism	77	1.6635	3	0.23061	1.467	1	1	0.30285
17	Ascorbate and aldarate metabolism	45	0.97216	2	0.25369	1.3716	1	1	0.03322
18	Primary bile acid biosynthesis	47	1.0154	2	0.26991	1.3097	1	1	0.06346
19	Glycine, serine and threonine metabolism	48	1.037	2	0.27802	1.2801	1	1	0.28435
20	Linoleic acid metabolism	15	0.32405	1	0.28005	1.2728	1	1	0.65625
21	Starch and sucrose metabolism	50	1.0802	2	0.29423	1.2234	1	1	0.00031
22	Pentose and glucuronate interconversions	53	1.145	2	0.31847	1.1442	1	1	0.08723
23	Taurine and hypotaurine metabolism	20	0.43207	1	0.35504	1.0355	1	1	0.08094
24	Pyrimidine metabolism	60	1.2962	2	0.37422	0.98292	1	1	0.09259
25	Thiamine metabolism	24	0.51849	1	0.40946	0.89291	1	1	0
26	Fatty acid elongation in mitochondria	27	0.5833	1	0.4473	0.80453	1	1	0
27	Pentose phosphate pathway	32	0.69132	1	0.50514	0.68292	1	1	0
28	Methane metabolism	34	0.73452	1	0.52658	0.64135	1	1	0
29	Amino sugar and nucleotide sugar metabolism	88	1.9011	2	0.57407	0.555	1	1	0
30	Butanoate metabolism	40	0.86415	1	0.58557	0.53517	1	1	0.0048
31	Purine metabolism	92	1.9875	2	0.59873	0.51294	1	1	0.00425
32	Phenylalanine metabolism	45	0.97216	1	0.62917	0.46335	1	1	0.11906
33	Fructose and mannose metabolism	48	1.037	1	0.65314	0.42597	1	1	0.02948
34	Porphyrin and chlorophyll metabolism	104	2.2468	2	0.6664	0.40586	1	1	0
35	Glyoxylate and dicarboxylate metabolism	50	1.0802	1	0.66826	0.40307	1	1	0.00686
36	Fatty acid metabolism	50	1.0802	1	0.66826	0.40307	1	1	0.02959
37	Arachidonic acid metabolism	62	1.3394	1	0.74634	0.29257	1	1	0.21669
38	Steroid hormone biosynthesis	99	2.1388	1	0.89008	0.11645	1	1	0.00391

**Figure 5:**
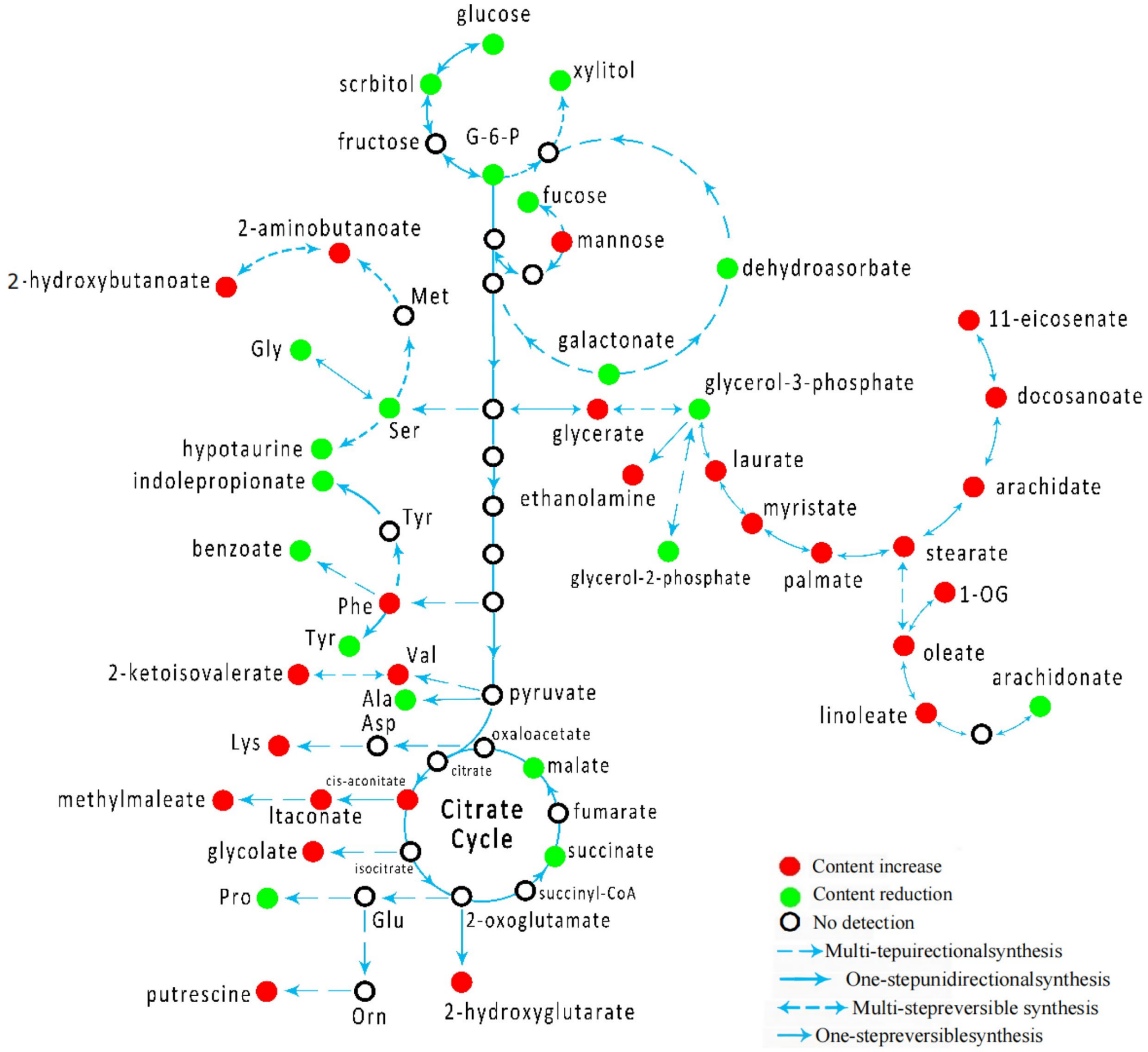
Schematic overview of Schematic overview of metabolic network in CSFV-infected group.

By establishing the connections between relational metabolic pathways, the metabolic networks in CSFV-infected groups were constructed (Figure 5).

### Correlation matrix of different metabolites between CSFV- and mock-infected groups at different times

**Figure 6:**
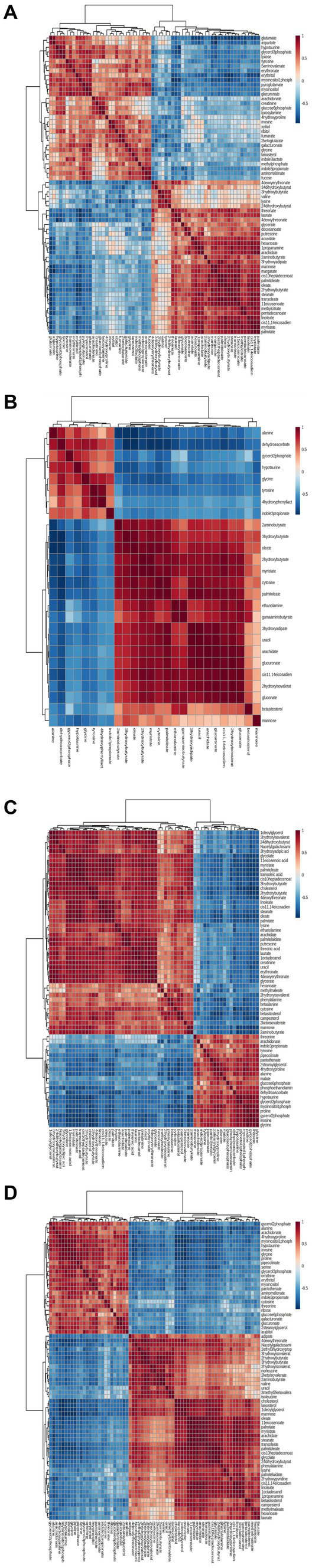
Correlation analyses of differentiated metabolic pathways between CSFV-infected and mg2 on 1DPI **(A)**, 3DPI **(B)**, 5DPI **(C)**, and 7DPI **(D)**, respectively.

To characterize the concentration correlations among different metabolites, we performed Pearson correlation analysis on the quantitative information of these substances, as shown in Figure 5. The rows and columns in the figure indicate these differences. The measured value of the correlation coefficient is shown on the right-hand side of the figure. The color depth of the square in the image is related to the correlation between the differences. From the color distribution, it can be seen that there is a high correlation between 1 DPI (Figure 6A), 3 DPI (Figure 6B), 5 DPI (Figure 6C), and 7 DPI (Figure 6D) and the concentration changes of metabolites with different metabolites in the same metabolic pathway or related functions in the mock-infected group, which indicated changes in metabolic pathways of different metabolites at all-time points after CSFV infection from the mock-infected group, rather than changes in individual metabolites.

### Heat map analysis of differential metabolites in CSFV- and mock-infected groups at different times

**Figure 7:**
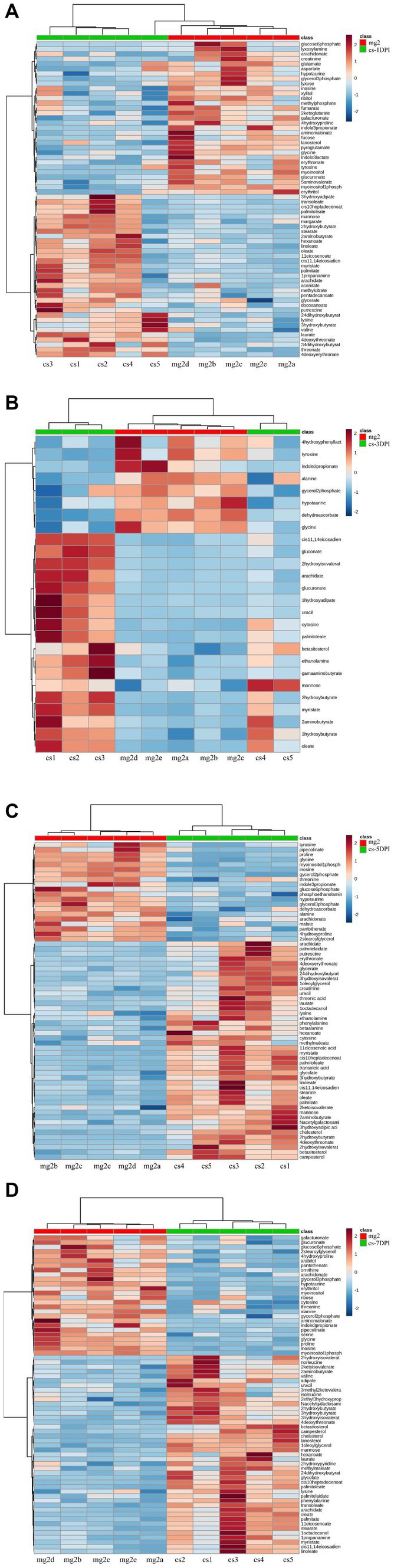
Heatmap of differentiated metabolic pathways between CSFV-infected and mg2 on 1DPI **(A)**, 3DPI **(B)**, 5DPI **(C)**, and 7DPI **(D)**, respectively.

To evaluate the relationship between the differential metabolites of CSFV- and mock-infected, we analyzed the quantitative information of these substances using a heatmap, as shown in Figure 6. Rows represent different metabolites, columns represent sample numbers, the upper tree structure represents the similarity relationship between samples, and the left tree structure represents the similarity clustering relationship between different metabolites. The analysis compared CSFV-infected groups cs-1DPI (Figure 7A), cs-3DPI (Figure 7B), cs-5DPI (Figure 7C), cs-7DPI (Figure 7D) with mg2. The different metabolites among the samples showed good similarity settlement relationships at all-time points after infection, which indicated that the intra-group differences in metabolic pathway changes caused by CSFV infection were small, and differences between the groups are obvious (Figures 7A–D).

## Discussion

The comparison of plasma metabolites between CSFV- and mock-infected groups showed significant differences in forty-five metabolites, among which twenty-five metabolites had higher abundance than those of the uninfected group and twenty metabolites had lower abundance than those of the mock-infected group. These metabolites were mainly distributed in the tricarboxylic acid cycle, amino acid cycle, sugar metabolism, and fat metabolism pathways. After CSFV infection, the tricarboxylic acid cycle, fatty acid biosynthesis, inositol phosphate metabolism, pentose and glucuronate interconversion, glycine, serine and threonine metabolism, linoleic acid metabolism, β-alanine metabolism, lysine metabolism, cyanoamino acid metabolism, pantothenate-CoA biosynthesis, aminoacyl-tRNA biosynthesis, arginine-proline metabolism, phenylalanine metabolism, linoleic acid metabolism, pantothenate metabolism, and arachidonic acid metabolism were altered. After CSFV infection, the contents of glucose 6-phosphate, xylitol, and fructose in sugar metabolism decreased, suggesting that CSFV infection has an impact on sugar metabolism. After CSFV infection, the production of malic acid and succinic acid in the tricarboxylic acid cycle was inhibited, and the metabolic pathway of cis-aconitic acid was increased, suggesting that CSFV infection affected the tricarboxylic acid cycle. CSFV infection also clearly affected nucleotide metabolism. On the one hand, CSFV infection promoted the metabolism of phenylalanine, valine, and lysine; on the other hand, CSFV infection inhibited the metabolism of serine, alanine, and tyrosine. Thus, CSFV infection leads to a disorder in lipid metabolism, in which the arachidonic acid metabolism pathway has a great influence.

Many viruses parasitize cells and cause diseases in the body, and almost all pathogens require help from their host metabolites to complete replication and reproduction. Metabolites differ from RNA and proteins. Rather than being directly encoded in the genome, metabolites are the products of biochemical pathways used by cells or tissues to promote their own survival or normal physiological metabolism. Using metabonomics to analyze the changes in metabolites in cells, tissues, or organisms can reveal the specific metabolites of some diseases and the different substances that provide potential metabolic pathways, which can offer a better understanding of the mutual mechanism between life and diseases. The distribution and changes in metabolites after virus infection provide the most direct evidence for the interaction between a host and pathogen; however, research on the differential analysis of metabolites between a host and virus is only in its infancy (12). With the rapid development of analytical techniques and pattern recognition methods, metabonomics will become a powerful tool for clarifying the complex interactions between hosts and pathogens. This approach can systematically evaluate and analyze low-molecular-weight metabolites with high throughput and identify characteristic biomarkers of diseases.

Because CSFV has an affinity for macrophages, endothelial cells (13, 14), and other auxiliary cells such as follicular dendritic cells (15), the virus may also use some metabolites of host cells to synthesize its own replication and reproduction. However, there has been little research on the differential analysis of metabolites *in vivo* and the changes in intracellular metabolites after CSFV infection using metabonomics to date. In this study, an animal model of experimental CSFV infection in Tibetan miniature pigs was selected, the metabolic substances in plasma infected by CSFV were dynamically studied, and the differences were analyzed by metabonomics for the first time.

The experimental data and results of the first part of this study confirmed that the animal model of CSFV infection was successfully established. Through dynamic detection of plasma metabolites in mock-infected and infected groups, we found 45 specific metabolites related to CSFV infection, which were mainly distributed in the metabolic pathways of the tricarboxylic acid cycle, amino acid cycle, sugar metabolism, and fat metabolism. The concentrations of 25 metabolites were higher than those of the mock-infected group and the concentrations of 20 metabolites were lower than those of the mock-infected group. In the model group after CSFV infection, various metabolites were upregulated or downregulated, indicating abnormal metabolism. The differential analysis of metabolites of CSFV infection can be inferred from the change in intermediates in the process of substance metabolism. Because the metabolites of CSFV infection are complex and diverse, some are related to various metabolic pathways. We can speculate on their roles in some metabolic pathways based on the differences between different metabolites. In addition, to describe the relative concentration change of CSFV target metabolites more clearly, we further analyzed the differences in metabolites between the infected and mock-infected groups. To study the influence of CSFV infection on different metabolic pathways, we compared the metabolic spectrum of CSFV-infected and mock-infected animals. The results showed that metabolites in plasma after CSFV infection caused changes to the tricarboxylic acid cycle, fatty acid biosynthesis, inositol phosphate metabolism, mutual conversion of pentose and glucose, glutamic acid, serine, and threonine metabolism, linoleic acid metabolism, β-alanine metabolism, lysine metabolism, cyanoamino acid metabolism, pantothenate-CoA biosynthesis, aminoacyl-tRNA biosynthesis, arginine and proline metabolism, phenylalanine metabolism, linoleic acid metabolism, and pantothenate metabolism. In particular, abnormal fatty acid biosynthesis was observed to occur at all-time points after CSFV infection.

Previous studies have shown that viral replication is closely associated with intracellular lipid metabolism (10, 16). HBV is an enveloped virus. To meet the needs of its own lipid membrane, it absorbs more external lipids from the host cells for envelope biosynthesis. Metabolic differences in patients with HBV infection are mainly manifested in the increase in lipid and glucose metabolism levels, and the decrease in lactate, alanine, valine, glutamine, and choline phosphate/choline levels. A recent study showed that HBV X protein can activate SREBP1 and PPAR to promote the biosynthesis of host lipids and then upregulate lipid synthesis in HBV (17, 18). Hepatitis B antigen may interfere with lipid metabolism in the liver (5, 18). It has also been reported that replication of HCMV enhances biosynthesis by inducing nucleic acids and proteins in host cells. HCMV infection stimulates glycolysis and the tricarboxylic acid cycle, and promotes pyrimidine synthesis (9). Saturated long-chain fatty acids are essential for the production of infected HCMV progenies (19). It has also been reported that both *Salmonella typhi* and *Salmonella* can use phospholipids in bile as important sources of carbon and energy (20). Dengue virus (DENV) infection also leads to the accumulation of long-chain fatty acids in the serum of patients (10). DENV infection redistributes fatty acid synthase to the viral replication site through the interaction between nonstructural protein 3 (NSP3) and fatty acid synthase, thereby promoting cellular fatty acid synthesis (21). Our study found that CSFV infection enhanced the biosynthesis of fatty acids, including the metabolism of linoleic acid and arachidonic acid. Among them, the synthesis of long-chain fatty acids, including lauric acid, myristate, palmitic acid, stearic acid, oleic acid, and linoleic acid, increased, whereas the metabolism of arachidonic acid decreased. The promotion of fatty acid biosynthesis by CSFV may promote the formation of a membrane virus replication complex and in turn facilitate virus transmission. Malic acid and succinic acid are intermediates of the tricarboxylic acid cycle in ATP, and their concentrations in the serum of infected pigs were lower than those in the mock-infected group, whereas the content of cis-aconitic acid was higher after infection than that in the mock-infected group. These findings indicate that the highly toxic CSFV Shimen strain inhibited the tricarboxylic acid cycle of energy production. The levels of phenylalanine and valine, representing the metabolism of amino acids, were higher than those in the mock-infected group, whereas the levels of serine, glycine, and tyrosine were downregulated after infection. Therefore, our results demonstrated that the metabolism of amino acids after CSFV infection was out of balance.

In summary, we investigated the pathogenic mechanisms of CSFV infection in an animal model using Tibetan miniature pigs. The results of metabonomics showed that many different metabolic pathways are altered after CSFV infection. Thus, the differential analysis of metabolites can play an important role in improving the diagnosis, prevention, and treatment of diseases (10). This study represents the first analysis of the changes in metabolites and metabolic pathways in plasma after CSFV infection, demonstrating that CSFV promotes viral replication and immune escape by regulating sugar metabolic pathways, amino acid metabolic pathways, the tricarboxylic acid cycle, and lipid metabolic pathways in the host. The metabonomics data of CSFV infection provided in this paper have important reference value for further study of CSF pathogenesis and the development of new diagnostic methods.

## Data availability statement

The original contributions presented in the study are included in the article/Supplementary material. Further inquiries can be directed to the corresponding author.

## Author contributions

JL, WH, and WW performed the experiments and wrote the manuscript. XW, SY, XN, WenZ, BZ, YS, WeiZ, and ZL analyzed the data. JC conceived and designed the experiments. All authors contributed to the article and approved the submitted version.
